# Skin levels of advanced glycation end products in correlation with periodontal conditions – a pilot study

**DOI:** 10.1186/s12903-026-08409-0

**Published:** 2026-04-24

**Authors:** M. Cyris, E. Högele, J. Wagner, B. Schulte, A. Zengin, M. Laudes, C. E. Dörfer, D. M. Schulte, C. Graetz

**Affiliations:** 1 Clinic of Conservative Dentistry and Periodontology, University Hospital of Schleswig-Holstein, Campus Kiel, Kiel, Germany; 2 Department of Oral and Maxillofacial Surgery, University Hospital of Schleswig-Holstein, Campus Kiel, Kiel, Germany; 3 Department for Internal Medicine I, University Hospital Schleswig-Holstein, Campus Kiel, Kiel, Germany; 4 Department of Medicine, School of Clinical Sciences at Monash Health, Monash University, Melbourne, VIC Australia; 5 Institute of Diabetes and Clinical Metabolic Research, University Hospital of Schleswig-Holstein, Campus Kiel, Kiel, Germany; 6 Division of Endocrinology, Diabetes and Clinical Nutrition, Department of Internal Medicine I, University Hospital Schleswig-Holstein, Campus Kiel, Kiel, Germany

**Keywords:** Diagnostic, Periodontitis, Diabetes, Cardiovascular risk, Advanced glycation end products, AGE Reader, Metabolism

## Abstract

**Background:**

Although associations between periodontitis and metabolic diseases are established, published data about the correlation of advanced glycation end products (AGEs) and periodontal inflammation are scarce. The aim of the pilot study was to assess the AGE-levels in the skin of patients with gingival (GI: bleeding on probing (BOP) positive, no periodontal pockets), or periodontal inflammation (PE + : BOP-positive with periodontal pockets), or individuals with a history of periodontitis but no signs of current inflammation (PE-: BOP-negative with clinical attachment loss) to evaluate a potential role of AGEs as a biomarker for disease severity and risk of metabolic comorbidities in patients with GI or PE.

**Methods:**

After an oral examination a total of 168 patients (healthy or with control systemic conditions e.g. diabetes) were classified into a GI (*n* = 68), PE + (*n* = 64) and PE- (*n* = 36) group. AGE levels were measured non-invasively on the skin of the inner forearm using a point-of-care AGE-reader. Cardiovascular (CV) risk was assessed based on numerical AGE read-out in the skin and diabetes risk was determined using the Finnish-Diabetes-Risk-Score (FINDRISC) questionnaire.

**Results:**

Significantly higher AGE levels were found in PE (2.1(0.6)) versus GI (1.7(0.6) (*p* < 0.001), whereas no significant association could be identified between the CV risk in PE versus GI (*p* = 0.196). A subgroup analysis in PE identified a positive association for patients with current gingival inflammation (PE +) and higher CV-risk (*p* = 0.031). The FINDRISC was higher in PE (10.1(5.1)) compared to GI (6.1(4.9)) (*p* < 0.001).

**Conclusion:**

Current inflammation in PE is associated with increased CV risk based on AGE levels of the skin. The AGE Reader may serve as a non-invasive screening tool in dental practice to support early CV-risk assessment and interdisciplinary prevention strategies.

**Trial registration:**

The study was retrospectively registered in the DRKS—German Clinical Trials Register (https://www.drks.de) with the registration-ID DRKS00031545 (23/03/2023).

## Introduction

Periodontitis is a chronic inflammatory disease with a multifactorial etiology and ranks as the sixth most common disease in the world [1]. One key risk factor is the dysbiosis of the oral biofilm that adheres to the tooth surface. Unless this is removed regularly, the host immune response can trigger an inflammatory reaction of the gingiva – known as gingivitis, the most common periodontal disease [2]. Gingivitis always precedes periodontitis, however, not every case of gingivitis progresses to periodontitis [3]. In gingivitis, the inflammation is confined to the gingiva and both the inflammation and tissue integrity are fully reversible, as there is no loss of connective tissue attachment or alveolar bone. In contrast, periodontitis [3] also involves inflammation that may be reduced or resolved with treatment, but the resulting destruction of the periodontal supporting tissues, including the alveolar bone [4], is irreversible and may ultimately lead to tooth loss.

Epidemiological data illustrate that, in addition to smoking, many metabolic diseases, such as diabetes mellitus (DM) significantly impact the inflammatory processes associated with gingivitis and periodontitis. The severity of gingival inflammation (GI) is closely linked to the degree of hyperglycemia [5]. Patients with both periodontitis and DM exhibit significantly higher probing depth (PD) and clinical attachment loss (CAL) compared to those without DM [6, 7]. Similarly, poorly controlled blood glucose levels have been associated with increased periodontitis progression and a higher risk of tooth loss compared to well-controlled blood glucose levels and non-diabetic patients [8].

Diabetes patients often develop microvascular and macrovascular complications. Frequently, they also suffer from concomitant diseases such as high blood pressure, dyslipidemia and obesity, all of which increase the risk of cardiovascular disease (CVD), which in turn is the leading causes of mortality worldwide and among diabetes patients [9].

Periodontitis is a chronic inflammatory condition driven by a dysbiotic biofilm and an exacerbated host immune response. Extensive epidemiological evidence links periodontitis with cardiovascular disease; while causality remains under investigation, shared inflammatory pathways and risk factors are well documented [10]. Nevertheless, it is important to note that causality has not yet been conclusively established [11].

The formation and accumulation of advanced glycation end products (AGEs) in various tissues represents one of the main pathogenetic mechanisms underlying diabetic complications. AGEs are irreversible adducts formed by non-enzymatic glycation of proteins and lipids with reducing sugars [12]. These compounds have proinflammatory effects on cells and can accumulate in tissues, including periodontal tissue. When AGEs bind to their signaling receptor RAGE [13], they can alter target cell function, leading to excessive inflammation and impaired tissue regeneration. It was shown years ago that PE can accelerate this process [14].

The interaction between AGEs and their receptor RAGE contributes not only to cellular oxidative stress and pro-inflammatory signaling but also to structural alterations within affected tissues. Activation of RAGE on epithelial and endothelial cells can induce thickening of basement membranes in mucosal and vascular structures [13]. This process impairs the diffusion of nutrients and immune mediators, thereby compromising tissue homeostasis and defence mechanisms. In periodontal tissues, these microstructural changes may exacerbate inflammation and delay repair, establishing a potential link between metabolic dysregulation and progressive periodontal destruction [14].

AGEs accumulate with age and metabolic imbalance, and their interactions with the receptor for AGEs (RAGE) triggers oxidative and inflammatory responses relevant to periodontal tissues. They also have been linked to several diseases, including cardiovascular disease, chronic kidney disease and autoimmune diseases [15]. In particular, AGEs contribute to myocardial and arterial stiffness, promoting the development of atherosclerosis [16]. Additionally, AGEs accumulate in the body throughout life, establishing a correlation between aging and AGE accumulation [17].

Non-invasive measurement of dermal AGEs via autofluorescence provides a feasible chairside method to assess long-term glycation status. It may offer early insight into systemic risk profiles relevant to dental patients.

The primary objective of this study was to compare dermal AGE levels across three groups stratified by clinical parameters: (1) [GI] individuals with gingival inflammation only (bleeding on probing (BOP) positive, no periodontal pockets), (2) [PE +] individuals with current periodontal inflammation (BOP-positive with periodontal pockets), and (3) [PE-] individuals with a history of periodontitis but no signs of current inflammation (BOP-negative with clinical attachment loss). The secondary objective was to explore associations between dermal AGEs, cardiovascular AGE-risk categories, and the Finnish Diabetes Risk Score (FINDRISC). We hypothesized that AGE levels are higher in PE compared with GI, and in PE + compared with PE −.

## Methods

### Study design and setting

This cross-sectional observational pilot study was conducted at the Clinic of Conservative Dentistry and Periodontology of the University Hospital Schleswig–Holstein, between September 2020 and January 2021.

### Participants

A total of 298 participants who met the inclusion criteria were recruited from the patient cohort hat attended the primary care unit of the clinic. Thus, the included participants represent a distinct clinical population, primarily composed of individuals with acute dental needs or referrals from external dental and medical practitioners to the university hospital. It should be noted that patient recruitment did not occur daily and was hindered by the COVID19 pandemic.

**Fig. 1:**
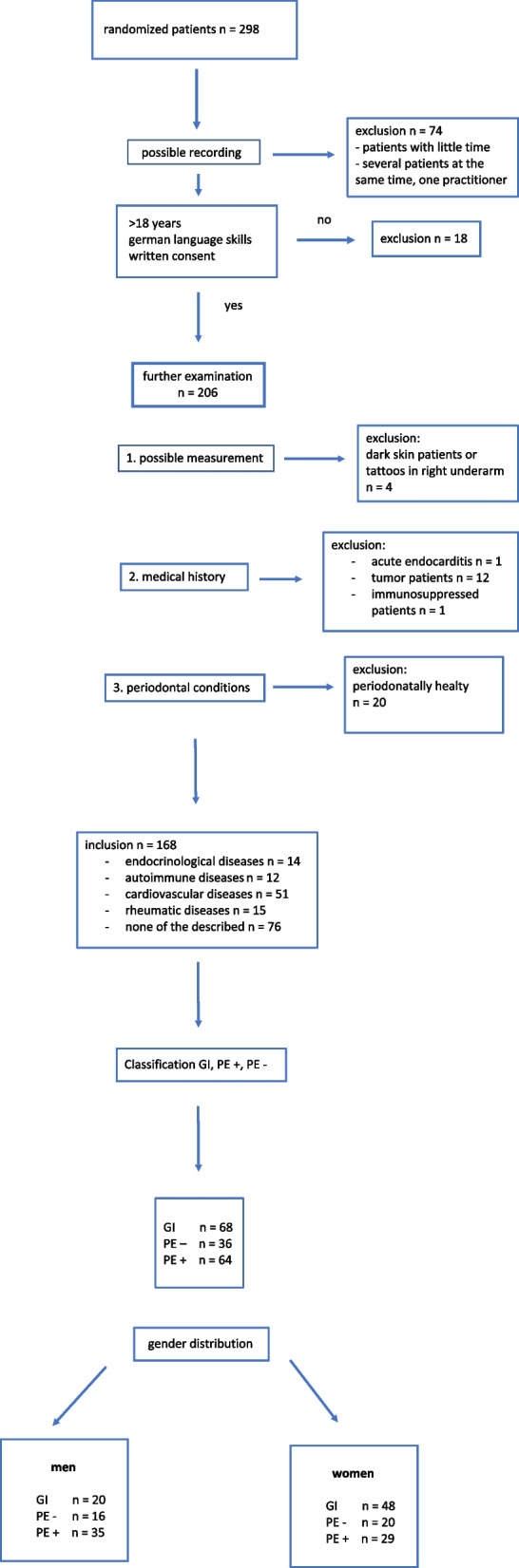
Flow chart of patient selection with inclusion and exclusion criteria and classification of patients into 3 groups (GI, PE-, PE +) with gender distribution. Values are shown as numbers (n)

To be eligible for inclusion, participants had to be ≥ 18years old, possess sufficient German language skills to independently complete the FINDRISC questionnaire and provide written informed consent for participation in the study (*n* = 168) (Fig. 1). All subjects completed a current medical history questionnaire, which included questions about heart disease, rheumatic diseases, liver, kidney and respiratory diseases, diabetes, infectious diseases and tumors.

Patients with controlled systemic diseases, such as endocrinological diseases (e.g. diabetes, thyroid diseases), autoimmune diseases (e.g. inflammatory bowel disease, Hashimoto's disease, multiple sclerosis, psoriasis), cardiovascular diseases (e.g. coronary heart disease, hypertension), and rheumatic diseases were included. However, patients with acute endocarditis, immunosuppressed patients, or tumor patients were excluded. Medical history was recorded using a standardized questionnaire; laboratory values such as glycated hemoglobin (HbA1c) were not measured. Self-reported control of chronic conditions was accepted. No laboratory test was performed as part of the dental treatment, consequently HbA1c and C-reactive protein (CRP) values are not available.

### Variables

The dependent variable was dermal AGE level (measured in arbitrary units via the AGE Reader). The independent variables included: Periodontal status via PSI (GI, PE-, PE +) and BOP, Age (in years), Sex (male/female), Body mass index (BMI) (kg/m^2^), Smoking status (current smoker: yes/no), FINDRISC (0–26 points, estimating 10-year diabetes risk), and Cardiovascular risk category (based on AGE value relative to age-adjusted reference ranges).

### Data sources and measurement

#### Periodontal examination

The intraoral examination assessed the number and condition of teeth, including the decayed, missing and filled teeth index (DMFT) [18, 19] as well as the periodontal screening index (PSI) [20] by using a special probe (WHO probe, E. Hahnenkratt GmbH) at six sites per tooth (mesiobuccal, buccal, distobuccal, mesiolingual, lingual, and distolingual) in sextants. The PSI scores range from code 0–4, reflecting the patient´s individual periodontal status. In addition to PSI scoring, BOP was recorded at all six sites per tooth. Thus, BOP data were available for all examined teeth and used together with the categorical probing pocket depth based on the PSI (e.g. ≤ 3.5 mm or ≥ 5.5 mm). Clinical examinations were performed by five dentists using a standardized protocol. All examiners received internal calibration training, and an inter-examiner measurement deviation of up to 1 mm was considered acceptable according to the clinic’s internal quality guidelines. Patients were classified into either the GI or PE group, whereby the PE group was further subdivided into one without current (PE-) and one with current (PE +) signs of inflammation. The classification was based on established criteria [21]. GI was defined as bleeding on probing (BOP) ≥ 10% with probing pocket depth (PPD) ≤ 3.5 mm and no attachment/bone loss. PE- was characterized by attachment/bone loss without current inflammation (BOP < 10%) and PPD ≥ 3.5 mm in contrast to PE +, which was defined in addition to attachment/bone loss and PPD ≥ 3.5 mm with current inflammation (BOP ≥ 10%).

However, in alignment with our primary aim to compare the extent of gingival or periodontal inflammation, we omitted a further detailed classification according to staging and grading. Likewise, the distinction between localized or generalized forms of periodontal inflammation was not applied. Although patients who exhibited a PSI code of 3 or higher in any sextant subsequently received a full-mouth attachment assessment as part of their further clinical management, these data were not included in the present analysis. The focus of this pilot study was to evaluate and compare two screening tools—the periodontal screening index (PSI) and the non-invasive AGE Reader—under conditions that reflect a simple and pragmatic screening approach suitable for clinical practice.

### AGE measurement

**Fig. 2:**
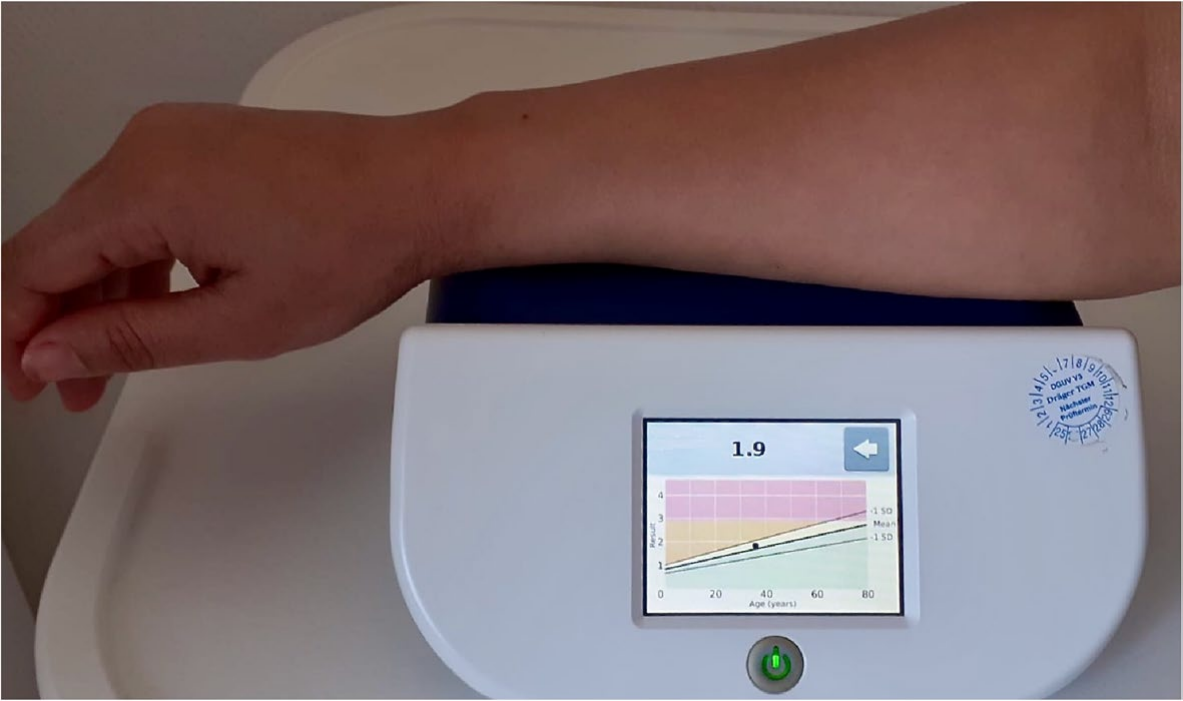
Non-invasive measurement of dermal advanced glycation end products (AGEs) using the AGE Reader (mu connect, Diagnoptics, Groningen, The Netherlands) on the inner side of the forearm

Additionally, a non-invasive assessment of dermal AGE level was conducted using an autofluorescence-based measurement with ultraviolet light (AGE Reader, mu connect, Diagnoptics, Groningen). Patients placed their clean right inner forearm on the AGE Reader spectrometer for a few seconds to obtain the AGE value automatically (Fig. 2).

The measured AGE levels were compared with reference values for healthy individuals, as AGE accumulation increases significantly with age.

**Fig. 3:**
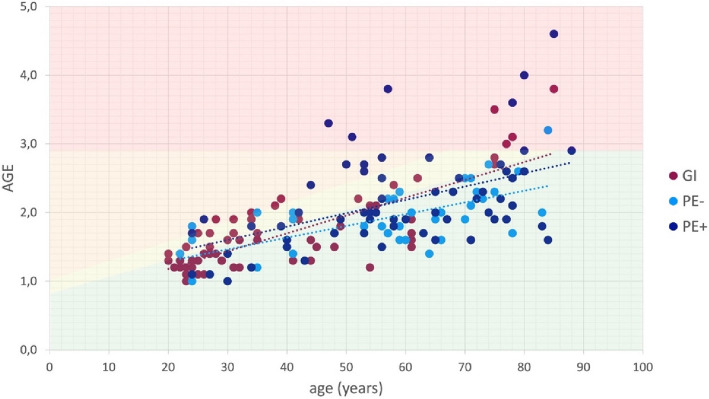
Illustration of cardiovascular (CV) risk in patients with gingival inflammation (GI) and patients with attachment loss with (PE +) and without (PE-) inflammation based on age and AGE values (green background: no CV risk; yellow background: slightly increased CV risk; orange background: increased CV risk; red background: clearly elevated CV risk)

The results were categorized into four CV risk groups using a color-coded system (see Fig. 3 for details):

- *Normal (no increased CV risk):* AGE value ≤ age-related mean.
- *Risk group I (slightly increased CV risk):* AGE value within one standard deviation of the age-related mean.
- *Risk group II (increased CV risk):* AGE value above one standard deviation of the age-related mean**.**
- *Risk group III (definite CV risk):* AGE value ≥ 2.9 [22].

### Systemic and behavioral factors

In addition we determined each individual's diabetes risk using eight questions from the FINDRISC questionnaire developed by the German Diabetes Foundation [23]. The questionnaire additionally contains questions addressing, among others, family predisposition, dietary and exercise habits, and the body mass index (BMI). The results of this questionnaire determine their individual DM type 2 risk in the next ten years using a point system. Whereby a score of less than 7, which represents a 1% risk, hardly corresponds to a risk of developing diabetes. The percentage of 1% means that one person in a hundred could develop type 2 diabetes in the next 10 years. At 7–11 points, a slight risk of 4% is present, 12–14 points correspond to an increased risk of 17%, 15–20 points indicate a high risk at 33%, and at over 20 points and a risk of 50%, there is an acute need for action [23].

Current smoking status was assessed by a standardized medical questionnaire and categorized dichotomously (yes/no response to: “Do you currently smoke?”).

### Statistical analysis

The statistical analysis of the measurements was performed with SPSS Statistics 28 (IBM, Chicago, IL, USA). Each individual participant served as the unit of analysis, with the AGE value being the primary response parameter of this study. The distribution of continuous variables was assessed using the Shapiro–Wilk test. Comparisons across the three study groups (GI, PE −, PE +) were performed using Kruskal–Wallis tests, followed by Bonferroni-adjusted pairwise Mann–Whitney U tests, as most variables were not normally distributed. For normally distributed variables, such as the FINDRISC, independent samples t-tests were applied to compare means between groups. All tests were two-sided; statistical significance was assumed if p ≤ 0.05. To explore potential confounding effects of age and AGE levels, we additionally conducted stratified analyses within relevant subgroups (e.g., by age group and AGE category), rather than applying formal multivariable regression models, due to the exploratory nature and limited sample size of this pilot study.

This investigation was designed as an exploratory pilot study, as no prior data were available on dermal AGE measurements in patients with different degrees of periodontal inflammation. Consequently, an a priori sample size calculation was not feasible due to the absence of comparable effect sizes or variance estimates in the literature. All eligible patients attending the dental clinic within the predefined study period and meeting inclusion criteria were consecutively enrolled, representing a pragmatic and unbiased recruitment approach. The resulting sample size of 168 participants provides valuable preliminary data to inform the design and power calculations of future confirmatory studies.

### Ethics approval and consent to participate

All procedures adhered to the ethical standards set forth by the institutional and national research committees (Kiel IRB: D447/20), as well as the 1964 Declaration of Helsinki and its subsequent revisions. Approval was granted by the ethics committee of the medical faculty of the Christian-Albrechts-University of Kiel. Before starting the study, verbal and written information about the study protocol was given to the participants and they provided written informed consent before enrolment. In addition, the study was retrospectively registered in the German Clinical Trials Register with the registration-ID DRKS00031545 (23/03/2023) as a non-interventional observational study.

## Results

### Participant characteristics

In total, data of *n* = 168 patients could be analysed concerning sex, age, number of teeth, BMI, AGE score, FINDRISC, cardiovascular risk and smoking status. Among them, 30 smokers were in the PE group (PE-/PE + : 10/20), while 14 were in the GI group. A total of 97 women and 71 men participated in the study (Fig. 1). The mean age of female participants was 44.2 years, while male participants had a mean age of 59.4 years. Demographic and clinical characteristics of the study population are shown in Table 1.

**Table 1:** Results of each group according to the study-based internal definition of gingival inflammation GI) and attachment loss without (PE-) and with signs of inflammation (PE +), as well as the total PE group

	**GI (*n* = 68)**	**PE- (*n* = 36)**	**PE + (*n* = 64)**	**PE total (*n* = 100)**	***p*-value**
N of women (%)	48 (70.6)	20 (55.6)	29 (45.3)	49 (49.0)	
N of men (%)	20 (29.4)	16 (44.4)	35 (54.7)	51 (51.0)	
age (yrs, mean)	39.4 ± 17.7 (20—85)	57.1 ± 18.0 (22—84)	58.9 ± 17.1 (24—88)	58.2 ± 17.4 (22—88)	< 0.001^x^**
N of < 50 (%)	49 (72.0)	10 (27.8)	18 (28.1)	28 (28.0)	
N of ≥ 50 (%)	19 (28.0)	26 (72.2)	46 (71.9)	72 (72.0)	
AGE (mean)	1.7 ± 0.6 (1–3.8)	1.9 ± 0.5 (1–3.2)	2.2 ± 0.7 (1–4.6)	2.1 ± 0.6 (1–4.6)	< 0.001^x^**
women	1.5	1.7	1.9	1.8	< 0.001^x^**
men	2.0	2.2	2.4	2.3	0.055^x^
FINDRISC^a^ (mean)	6.1 ± 4.8 (0—20)	9.6 ± 5.7 (0—23)	10.3 ± 4.6 (1—23)	10.1 ± 5.0 (0—23)	< 0.001^y^**
teeth^b^ (mean)	25.5 ± 4.7 (2—28)	24.7 ± 4.1 (11—28)	22.4 ± 5.9 (1—28)	23.3 ± 5.4 (1—28)	< 0.001^x^**
N of subjects < 20 teeth (%)	6 (8.8)	4 (11.1)	19 (29.7)	23 (23.0)	
N of subjects 20 to 25 (%)	12 (17.7)	12 (33.3)	18 (28.1)	30 (30.0)	
N of subjects > 25 (%)	50 (73.5)	20 (55.6)	27 (42.2)	47 (47.0)	
BMI (kg/m^2^, mean)	25.0 ± 4.6 (19—34)	25.3 ± 3.7 (18—40)	27.2 ± 4.3 (20—40)	26.5 ± 4.2 (19—40)	0.014^x^*
N of subjects < 25 (%)	33 (48.5)	14 (38.9)	17 (26.6)	31 (31.0)	
N of subjects 25–30 (%)	29 (42.7)	20 (55.6)	35 (54.7)	55 (55.0)	
N of subjects > 30 (%)	6 (8.8)	2 (5.5)	12 (18.7)	14 (14.0)	
Cardiovascular risk N of subjects CV 0 (%)	43 (63.2)	26 (72.2)	41 (64.1)	67 (67.0)	
N of subjects CV 1 (%)	19 (28.0)	9 (25.0)	8 (12.5)	17 (17.0)	
N of subjects CV 2 (%)	2 (2.9)	0 (0)	9 (14.1)	9 (9.0)	
N of subjects CV 3 (%)	4 (5.9)	1 (2.8)	6 (9.3)	7 (7.0)	
N of smoker (%)	14 (20.6)	10 (27.8)	20 (31.2)	30 (30.0)	0.175^x^
N of women (%)	10 (20.8)	5 (25.0)	7 (24.1)	12 (24.5)	0.669^x^
N of men (%)	4 (20.0)	5 (31.2)	13 (37.1)	18 (35.3)	0.231^x^

Values are presented as numbers (n) and mean values

*P*-value represents the comparison between GI and PE total

*AGE* Advanced glycation end products, *BMI* Body mass index, *CV* Cardiovascular risk

ᵃmax. 26ᵇ max. 28 ^**×**^ Wilcoxon—Mann – Whitney – Test ^**y**^ T-Test * significant ** highly significant

### Periodontal variables

The GI group included 68 patients and the PE group 100 patients, subdivided into PE + (*n* = 64) and PE—(*n* = 36) (Table 1). On average, patients in the GI group had 25.5 teeth, while those in the PE group had 23.3 teeth (PE-/PE + : 24.7/22.4), based on a full dentition of 28 teeth. The GI group contained more women than men (male/female: 20/48) and in the PE group the gender distribution was approximately equal (male/female: 51/49) (Fig. 1). The mean age in the GI group was 39.4 years, compared to 58.2 years in the PE group, while the mean age in PE- was 57.1 years and in the PE + 58.9 years.

### Dermal AGE levels

AGE levels were lower in gingivitis patients than in PE patients (GI/PE: 1.7/2.1). Within the PE subgroup, AGE values were 1.9 for PE- and 2.2 for PE +. A gender comparison shows that men have higher AGE value across all groups (GI: 2.0/1.5; PE-: 2.2/1.7; PE + : 2.4/1.9; PE total: 2.3/1.8). No significant association was found between cardiovascular risk score in PE (0.56(0.93)) and GI (0.51(0.82)) groups (*p* = 0.196, see Table 1). Subgroup analysis in PE revealed a positive association between patients with current inflammation (PE + : 0.69(1.04) vs. without (PE-: 0.33(0.63) and a higher CV-risk score (*p* = 0.031) (Fig. 3).

### Associations with systemic parameters

**Fig. 4:**
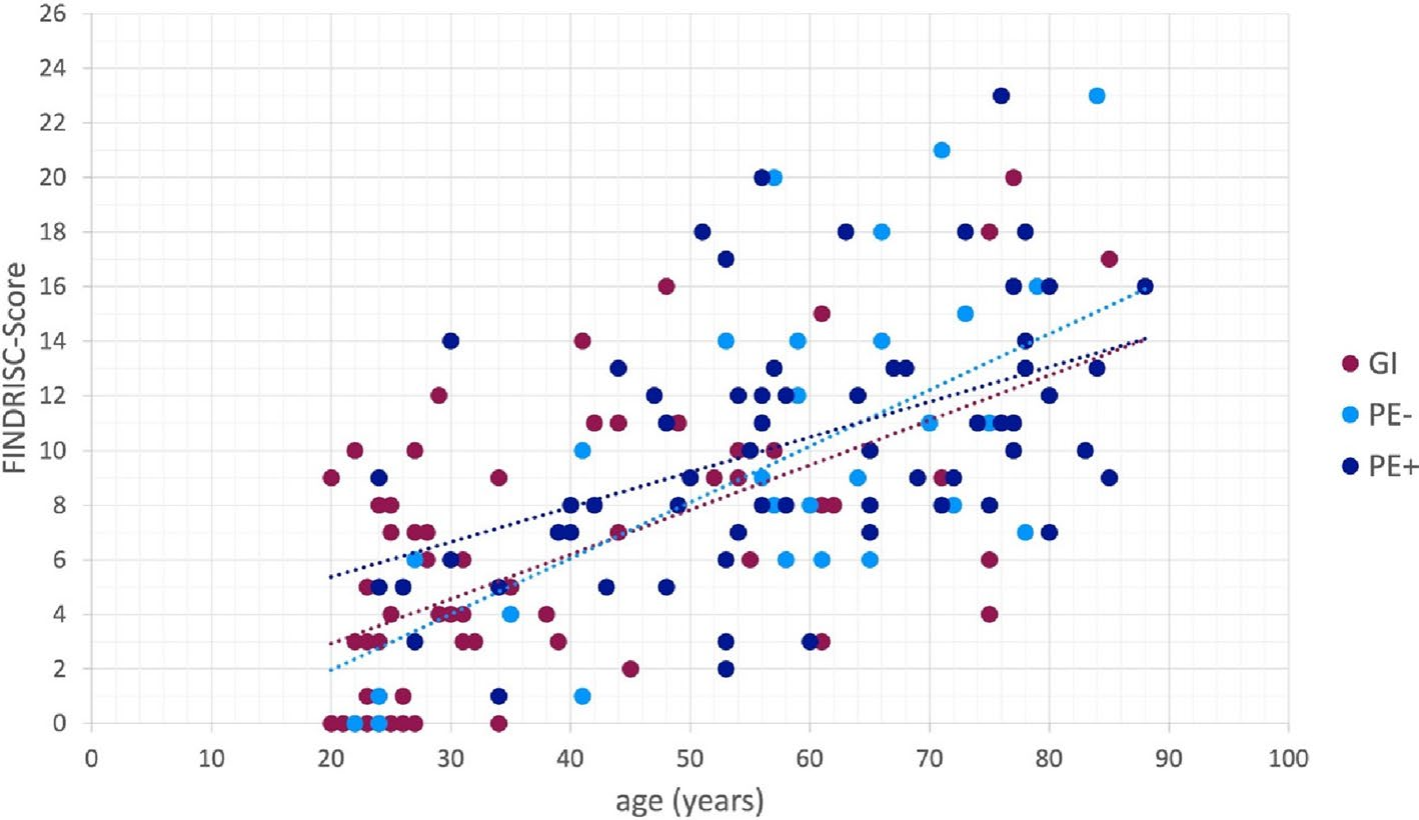
Influence of periodontal diagnosis gingival inflammation (GI) (*n* = 68) and attachment loss (*n* = 100) with (PE +) and without (PE-) inflammation) on the association between FINDRISC (minimum = 0, maximum = 26) and age

Patients in the PE group had a higher FINDRISC than those in the GI group (PE/GI:10.1/6.1) (Fig. 4). Additionally, the PE-group had a higher mean BMI (27 (GI: 25) *p* = 0.014). After adjusting for AGE levels and age, 110 patients showed no CV-risk (GI/PE: 43/67), 36 had a slightly elevated CV-risk (GI/PE: 19/17), 11 had an increased CV-risk (GI/PE: 2/9) and 11 patients had a definite CV-risk (GI/PE: 4/7).

## Discussion

In this cross-sectional pilot study, dermal AGE levels were significantly higher in participants with periodontitis (PE) than in those with gingivitis (GI). Within the PE group, participants showing signs of current inflammation (PE +) exhibited higher AGE-based cardiovascular risk categories than those without active inflammation (PE −).

Diabetes has become a global health concern in recent years, with a significant increase in its prevalence worldwide [24]. It is associated with various complications, particularly vascular disorders such as atherosclerotic cardiovascular or peripheral vascular disease, which contribute substantially to diabetes-related mortality [25]. The well-established bidirectional relationship between diabetes and periodontitis further highlights the importance of identifying shared biomarkers to assess the risk of the PE, DM and CVD [14]. Moreover, obesity is a well-known major risk factors for type 2 diabetes, particularly in industrialized countries. In our study 104 patients had a BMI of 25 and higher classifying them as overweight [26]. Chronic obesity and the subsequent development of type 2 diabetes increase the risk of bidirectional relationships with other diseases, such as periodontitis. These conditions, in turn, can lead to metabolic complications, making the measurement of AGE levels a potentially valuable tool for risk assessment and identification of high-risk patients [27].

To our knowledge, this is the first study to show that higher CV-risk score, based on dermal AGE levels measured non-invasively, is associated with gingival inflammation in periodontitis. In detail, we found no significant difference for the CV-risk between the GI and PE groups (*p* = 0.196), subgroup analysis of PE patients revealed a higher CV-risk in those with current inflammation (PE +), indicating increased inflammatory parameters (*p* = 0.031). This suggests a positive correlation between elevated dermal AGE levels and current inflammation in patients with periodontitis of PPD ≥ 3.5mm and BOP ≥ 10%.

Beyond these direct associations, periodontal and systemic diseases share a number of common risk factors. Lifestyle-related determinants such as poor diet quality, smoking, and chronic psychosocial stress contribute to both periodontal and systemic inflammation by promoting oxidative stress and AGE formation. This “shared risk factor” concept provides a broader pathophysiological context for our findings, suggesting that elevated dermal AGE levels may reflect the cumulative burden of metabolic and behavioral stressors rather than a disease-specific pathway. Integrating lifestyle modification strategies into preventive dental care may therefore have systemic benefits extending beyond oral health [28].

The AGE Reader used in this study is a non-invasive device that measures AGE levels in the skin by detecting skin autofluorescence emitted by AGEs. Several factors may influence these measurements, including diet, smoking habits, skin type [15] and alterations in blood flow [29]. The device offers multiple advantages: it is compact, easy to use and highly mobile due to battery operation, making it suitable for various settings such as nursing homes, workshops for individuals with disabilities and facilities for non-mobile patients. Measurements are uncomplicated, painless, quick and can aid in the early detection of CV or DM. It has already been shown that skin autofluorescence measurement enhances the predictive power of the FINDRISC for diabetes detection [30].

However, the AGE Reader has notable limitations. Initially designed for risk assessment of CV and DM, it was not specifically intended for dental applications [31]. A major drawback is its restricted use in individuals with darker skin tones (skin phototype V-VI), which limits its applicability in diverse populations [15]. Additionally, in our current pilot study, the AGE Reader could not accurately measure AGE levels in individuals with tattoos in the measurement area.

Unlike routine dental examinations, which patients typically attend up to twice a year, Germany currently lacks a standardized screening program for CV risk [32]. Since the AGE Reader can be used, among other things, to assess the risk of CV, it is precisely this advantage of regular dental recall that can be exploited by referring patients to the appropriate specialist in the event of a conspicuous AGE value. AGE accumulation is known to increase with age, smoking, and obesity—all established risk factors for both periodontitis and cardiometabolic disease. While group differences in AGE levels persisted after adjustment for these covariates, residual confounding cannot be excluded.

In addition to AGE measurement, we assessed each patient´s risk of developing type 2 diabetes using the FINDRISC questionnaire. Li et al. showed that this tool is effective in identifying undiagnosed diabetes within the German population [33]. In addition, it was shown a correlation between diabetes progression and the FINDRISC [34].

Our findings revealed that the cohort of patients suffering from gingivitis had an average score of 6.1 and the cohort of patients suffering from periodontitis had an average score of 10.1 (*p* = < 0.001). PE- had a mean score of 9.6 and PE + had the highest mean score of 10.3. Based on these results, GI patients in our cohort had a low 1% risk of developing type 2 diabetes, whereas periodontitis patients – regardless of inflammation status—had a slightly elevated risk of 4%. Despite the modest risk increase, our results support a positive association between type 2 diabetes and periodontitis.

Early identification of periodontitis patients with an elevated diabetes risk could provide an opportunity for timely lifestyle modifications, such as dietary improvements and increased physical activity, which have been shown to prevent or delay diabetes onset [34]. In case of a high identified risk, the affected Patients with high FINDRISC could also be referred to specialists for further evaluation. Since the FINDRISC questionnaire is simple to use and can be completed alongside routine medical history intake, it could serve as a valuable tool in dental practice for rapid diabetes risk assessment. This, in turn, could foster better interdisciplinary collaboration between dentists, general practitioners and internists.

The majority of the study participants were women (*n* = 97), 64% of whom were under 50 years old, while 79% of the male participants (*n* = 71) were over 50. This aligns with findings in Germany, where women are significantly more likely to seek dental treatment than men, who tend to seek service later in life [35]. In addition, our results showed that while women were equally distributed between the GI (*n* = 48) and PE (*n* = 49) groups, men had a higher prevalence of PE (*n* = 51) compared to GI (*n* = 20). If we attribute this to more frequent dental visits by women, then it can be concluded that periodontal problems, among others, can be diagnosed earlier in women and treated accordingly [36]. Therefore, our results must be interpreted with caution as we were not able to control for balanced gender or age distribution in our outpatient clinic during recruitment. Women are known to follow healthier diets than men, consuming more fruits, vegetables and whole grains [37], foods associated with lower AGE levels. It should also be recognized that the cohort of female patients, 64% of whom were under 50 years of age, was significantly younger than the male cohort, 79% of whom were over 50 years of age, and it must not be neglected in the interpretation that, as already mentioned, the AGE value is correlated with age and is therefore higher with age [17].

Our findings also confirm the well-established association between smoking and periodontal disease, with smokers exhibiting an 85% increased risk [38]. In our study, smoking prevalence was significantly higher in the PE group (56%) than in the GI group (21%), with the highest prevalence in the PE + group (69%). Smoking is also a known risk factor for cardiovascular disease [39] and doubles the risk of developing type 2 diabetes [40].

### Study limitations

Since the recruitment for this pilot study included all patients presenting for dental treatment in our outpatient clinic over a predefined period, we were not able to control for selection bias, like age or gender distribution. Furthermore, no statement can be made regarding the accuracy of responses on the FINDRISC questionnaires, as participants often did not know their own weight or were uncomfortable discussing it.

There were also certain limitations in the classification of patients into the GI, PE- and PE + groups to overcome. Many of the patients were already known to us and had data such as the periodontal status and X-rays. However, there were also some patients either lacking X-rays or declined them, requiring categorization based solely on clinical findings Therefore, we decided not to investigate the stage and grade of PE in detail, and performed the data analysis on inflammation present in GI or PE. This must be acknowledged as a major limitation, although it provided a feasible approach to simplify diagnosis according to the 2018 classification in this pilot study.

Periodontal measurements were performed using a WHO probe, which allows categorical recording of probing depths but not continuous millimetre values. This limitation may have introduced minor imprecision in distinguishing moderate from severe pocket depths. No formal examiner calibration dataset was available, and the relatively small sample size limits generalizability. Finally, the cross-sectional design precludes causal inference. Another limitation of this study is that systemic health information, including diabetes status, was based on self-reported medical history rather than laboratory confirmation. Therefore, parameters such as HbA1c were not available to objectively verify glycemic control. Although only patients who reported stable or medically controlled systemic conditions were included, the possibility of underreporting or misclassification cannot be excluded.

Due to the exploratory design and limited sample size, we did not conduct multivariable regression analyses. Potential confounding by age and AGE levels was instead addressed through stratified analyses within relevant subgroups. While this provides preliminary insight, it does not fully account for residual confounding.

While many studies investigated the correlation of AGE levels in DM and other associated comorbidities, the correlation between PE and AGE remains limited and definitely needs further research in the future. Future studies should include a larger cohort of patients with better control of risk factor (e.g. smoking, aging) for both diseases PE and DM.

## Conclusions

Within the study limitations, our results indicate dermal AGE levels were higher in patients with current periodontal inflammation and were associated with indicators of current gingival inflammation. Non-invasive AGE measurement may provide a simple adjunctive tool for identifying individuals with an unfavourable systemic risk profile in dental practice.

## Data Availability

The datasets used and/or analyzed during the current study are available from C. Graetz or D.M. Schulte on reasonable request.

## Abbreviations

AGE: Advanced Glycation End Product
GI: Gingival inflammation
PE: Periodontal inflammation
CV: Cardiovascular
DM: Diabetes Mellitus
CVD: Cardiovascular Disease
BMI: Body Mass Index
FINDRISC: Finnish Diabetes-Risk-Score
PSI: Periodontal Screening Index
BOP: Bleeding on Probing
HbA1c: Haemoglobin A1c value respectively glycosylated haemoglobin
